# Supplementary material for: A workflow with R: Phylogenetic analyses and visualizations using mitochondrial cytochrome b gene sequences
Source: PLoS One. 2020 Dec 15;15(12):e0243927. doi: 10.1371/journal.pone.0243927 (PMC7737995; doi:10.1371/journal.pone.0243927)
Supplement: Attachment — Submitted filename: Response-to-Reviewers.docx [file pone.0243927.s003.docx]

Dear Dr Brewer,

We have now completed our revisions, we thank reviewers for their constructive comments and contributions. Below our responses to reviewers are highlighted in color.

Best regards,

Dr Ugur Bilge

1. Please ensure that your manuscript meets PLOS ONE's style requirements, including those for file naming. The PLOS ONE style templates can be found at

<https://journals.plos.org/plosone/s/file?id=wjVg/PLOSOne_formatting_sample_main_body.pdf> and

<https://journals.plos.org/plosone/s/file?id=ba62/PLOSOne_formatting_sample_title_authors_affiliations.pdf>

Yes, checked the requirements.

Reviewers' comments:

Reviewer's Responses to Questions

**Comments to the Author**

1. Is the manuscript technically sound, and do the data support the conclusions?

The manuscript must describe a technically sound piece of scientific research with data that supports the conclusions. Experiments must have been conducted rigorously, with appropriate controls, replication, and sample sizes. The conclusions must be drawn appropriately based on the data presented.

Reviewer #1: Yes

Reviewer #2: Yes

2. Has the statistical analysis been performed appropriately and rigorously?

Reviewer #1: Yes

Reviewer #2: Yes

3. Have the authors made all data underlying the findings in their manuscript fully available?

The [PLOS Data policy](http://www.plosone.org/static/policies.action#sharing) requires authors to make all data underlying the findings described in their manuscript fully available without restriction, with rare exception (please refer to the Data Availability Statement in the manuscript PDF file). The data should be provided as part of the manuscript or its supporting information, or deposited to a public repository. For example, in addition to summary statistics, the data points behind means, medians and variance measures should be available. If there are restrictions on publicly sharing data—e.g. participant privacy or use of data from a third party—those must be specified.

Reviewer #1: Yes

Reviewer #2: Yes

4. Is the manuscript presented in an intelligible fashion and written in standard English?

PLOS ONE does not copyedit accepted manuscripts, so the language in submitted articles must be clear, correct, and unambiguous. Any typographical or grammatical errors should be corrected at revision, so please note any specific errors here.

Reviewer #1: No

We had the manuscript proofread by a native English speaker.

Reviewer #2: Yes

5. Review Comments to the Author

Please use the space provided to explain your answers to the questions above. You may also include additional comments for the author, including concerns about dual publication, research ethics, or publication ethics. (Please upload your review as an attachment if it exceeds 20,000 characters)

**Reviewer #1:** This manuscript presents exciting and beneficial research for computational phylogenetics, and should be published.

Thank you very much for your kind words and encouragement.

There are grammatical issues throughout the manuscript which should be addressed, and it may be advisable to enlist the aid of a dedicated copyeditor.

Yes, we got the manuscript proofreaded by a native English speaker.

While fairly straightforward, I do think that Figure 1 needs a descriptive caption to convey what you want the reader to take away from the figure.

Thank you for your suggestion. In line with your advice, we added a more descriptive caption for Figure 1 to lines 314-318 in the Manuscript.docx file.

Likewise, the caption for Figure 3 discusses which commands were used to construct the figure, and while the purpose of the figure here is to demonstrate the utility of these commands, it would still be advisable to provide some explanation of what information the figure itself is meant to convey. For example, what do the vertical hash marks indicate? Does the variation in branch length tell us anything? You convey these things in the caption for Figure 4, but if you are going to only explain them in one figüre why not do so in the first figure of this type which the reader will see?

Thank you for your warning and advice. In lines 369:374, Figure 3 caption (lines 377:381), and Figure 4 caption (lines 384:389) of the Manuscript.docx file, we added more information about what the vertical hash marks indicate and what the variation in branch length tells us.

For Figures 5 and 6, it would be good to put "site substitutions" after the 0.006 over the scale bar, for clarity.

Thank you so much for your suggestion. We added "site substitutions" after 0.006 over the scale bar placed in Figures 5 and 6 in line with your advice.

For the most part, the walkthrough is well written and easy to follow. However, I do have some comments:

In the walkthrough, you have readers install the "stats" package twice.

Thank you very much! One install.packages("stats") have been deleted.

It would be good to make sure that the instructions in the walkthrough are written in such a way so that people do not just stop reading when they get to, for example, "If your arrays are already aligned, please proceed from "READING AND PLOTTING OF ALIGNMENT-NJ-MSAPLOT" section" and miss important instructions in the same paragraph. The above quote is followed by a general warning of how long the alignment can take, which is then followed by an important instruction about calling the previously aligned file to the RStudio console. Make sure the walkthrough is written to be useable by people with little to no knowledge of R. Your code is helpful and you want lots of people to use it, so the lower you can make the bar to entry the better.

We are grateful, thank you for your advice. This perspective is very important to us. As you indicated, we would like to create a helpful protocol for anyone who wants to use it. Therefore, we added detailed information that can be read step by step in the S1_Appendix.txt file. Additionally, we also added some information about alignment time in S1_Appendix.txt file. We are hopeful that it would be more understandable, helpful, and useful in its final form.

Along these same lines, the walkthrough is written such that even if I do not need to align my sequences (as an example), I still need to perform some of the commands in the sequence alignment section in order to complete tasks described further down in the walkthrough. This could be another barrier to use of your code by those not well versed in R.

Thank you for your recommendations. We checked again all of these codes in line with your suggestions. We separated the beginning of codes as two for the aligned and non-aligned file. The section between "If (AlignNeeded)" and "export.fasta ()" in the S1_Appendix.txt file can only be used by people who want to align their sequences using the msa package. For already aligned files, the walkthrough will be available without issues.

Unfortunately, I was unable to test your code using the, relatively, small .fas files which I use for my research. Your code worked fine when tested with your example fsa file, but my 124 taxa, 1016 bp .fsa file caused RStudio to crash when I used nbin<-fasta2DNAbin(fname). I have not had trouble importing these data into RStudio in the past, so I assume it has something to do with your code. If your code cannot handle sequences of over 1000 bp, it should be noted in the publication. I was able to test your code on a ~500 bp, 137 taxa .fsa file, however, so your code works for shorter sequences.

Thank you so much you for your warnings and advice. We noticed a few important points and checked them again. We tested again 740 samples whose length is 1226 bp by following the steps. Also, another aligned FASTA file (43 sample, 1231 bp) was analyzed using the codes. They worked well after checking. If you would like to contact us, we would be very happy to test your sequences. Thus we can understand where there is a problem in codes as this can be a very important point.

**Reviewer #2:** I found this manuscript to have a very helpful purpose: providing a worked example of population genetic and phylogenetic analysis in R. The manuscript was mostly clearly written. Below I outline a few key areas for improvement.

Thank you very much for your encouragement.

First a few more general suggestions for improvement:

1) There is nothing specific to RStudio in this workflow. I would encourage the authors to simple find and replace all instances of "RStudio" with "R". The section explaining that RStudio is a popular IDE could optionally remain. I suggest this because the authors do not discuss any RStudio-specific functionality, so continually referring to RStudio is misleading or distracting. I also make this suggestion because there remains a substantial group of R users who are uninterested in adopting RStudio for their work, but who would still find the contents of this manuscript useful.

Thank you very much for your suggestions. We replaced "RStudio" instances with "R" in the Manuscript.docx file. Additionally, we would like to specify, all of the codes had been constructed in RStudio and tested in RGUI. At this stage, we noticed some advantages of RStudio that can be useful for researchers who are beginners in R or researchers who study visualization of analysis such as network, mapping etc. Therefore, we added “and RStudio” in line 15 and we added some advantageous information about RStudio in line 437:440 of the Manuscript.docx file for the reader and user RStudio.

2) The authors repeatedly highlight that some returned objects are of the S4 class system. However, the authors do not explain what is significant about S4 classes, nor how they compare to the S3 and R5 systems, nor any potential enter-operability concerns among different class systems. Thus I would suggest either including more information about S4 and why it matters to the analyses, or I would encourage the authors to simply remove any reference to S4.

Thank you very much for your suggestion. In line with your advice, we removed "S4" word in the Manucript.docx file.

3) The code examples in the main text need to be properly formatted. As they stand now, they are extremely hard to read. The main issue is a lack of proper line breaking and indentation. R Markdown or Sweave could be used to produce the proper formatting.

Thank you very much for your warning and advice. We output R Markdown document (.rmd file) and convert to MS Word document (.docx file) by using RStudio and added again the code examples in the Manuscript.docx file. We preferred to use syntax highlighting for easier understanding of the arguments in the sample codes.


Finally, here are a few edits for increased clarity:

lines 58--60: remove the sentence about Sweave, starting "Sweave, a powerful..." and ending "...and calculation results [10]." Sweave is never mentioned again, and so does not need to be introduced here. It is probably also no longer the first choice of most R users for making dynamical documents as most people now seem to use R Markdown and knitr.

Thank you very much for your warnings. We removed the sentence about Sweave, starting "Sweave, a powerful..." and ending "...and calculation results [10] in lines 58--60. We added information about Markdown, R Markdown and knitr package in lines 34:39 of Manuscript.docx. file.

lines 62--66: remove the sentences beginning "R also can be used..." and ending "...and store data analysis [10]." The purpose of this section is unclear and the use of the term "environment" (a very specific term in R) is incorrect in the technical context of R programming.

Thank you very much for your advice. We removed the sentences beginning "R also can be used..." and ending "...and store data analysis [10] in lines 62:66.

line 93: the semicolon (;) should be a colon (:)

Thank you very much for the correction. The semicolon (;) in line 61 of Manuscript.docx file had been replaced with a colon (:).

line 98: delete "array"

Thank you very much for your advice. We deleted “array” in line 98.

line 180: instead of saying "S4 class" saw what the actual class name is

Thank you very much for your advice. We removed "S4 class" in lines 176:177.

line 207: "subtracted" should be "extracted"

Thank you very much for your advice. We replaced "subtracted" with "extracted" in line 175 of Manuscript.docx. file.
