# Supplementary material for: A workflow with R: Phylogenetic analyses and visualizations using mitochondrial cytochrome b gene sequences
Source: PLoS One. 2020 Dec 15;15(12):e0243927. doi: 10.1371/journal.pone.0243927 (PMC7737995; doi:10.1371/journal.pone.0243927)
Supplement: Attachment — Submitted filename: Response-to-Reviewers.docx [file pone.0243927.s004.docx]

Dear Dr Brewer,

We promptly completed the minor revision, we thank reviewer for their constructive comments and contributions. Below our responses to reviewer are highlighted in color.

Best regards,

Dr Ugur Bilge

**Reviewers' comments:

Reviewer's Responses to Questions**

**Comments to the Author**

1. If the authors have adequately addressed your comments raised in a previous round of review and you feel that this manuscript is now acceptable for publication, you may indicate that here to bypass the “Comments to the Author” section, enter your conflict of interest statement in the “Confidential to Editor” section, and submit your "Accept" recommendation.

**Reviewer #1:** All comments have been addressed

Thank you!

2. Is the manuscript technically sound, and do the data support the conclusions?

The manuscript must describe a technically sound piece of scientific research with data that supports the conclusions. Experiments must have been conducted rigorously, with appropriate controls, replication, and sample sizes. The conclusions must be drawn appropriately based on the data presented.

**Reviewer #1:** Yes

3. Has the statistical analysis been performed appropriately and rigorously?

**Reviewer #1**: N/A

4. Have the authors made all data underlying the findings in their manuscript fully available?

The PLOS Data policy requires authors to make all data underlying the findings described in their manuscript fully available without restriction, with rare exception (please refer to the Data Availability Statement in the manuscript PDF file). The data should be provided as part of the manuscript or its supporting information, or deposited to a public repository. For example, in addition to summary statistics, the data points behind means, medians and variance measures should be available. If there are restrictions on publicly sharing data—e.g. participant privacy or use of data from a third party—those must be specified.

**Reviewer #1:** Yes

5. Is the manuscript presented in an intelligible fashion and written in standard English?

PLOS ONE does not copyedit accepted manuscripts, so the language in submitted articles must be clear, correct, and unambiguous. Any typographical or grammatical errors should be corrected at revision, so please note any specific errors here.

**Reviewer #1:** Yes

6. Review Comments to the Author

Please use the space provided to explain your answers to the questions above. You may also include additional comments for the author, including concerns about dual publication, research ethics, or publication ethics. (Please upload your review as an attachment if it exceeds 20,000 characters)

**Reviewer #1:** This is a much improved manuscript and will be a wonderful contribution to the ability of researchers to efficiently and effectively analyze and visualize phylogenetic data.

Thank you very much for your kind words and encouragement.

I do have a few very minor grammatical points:

**Line 433:** "and" needed after "tree type,"

We did it.

**Line 435:** This sentence is a bit clumsy, perhaps reword to "While input-output files are frequently needed in phylogenetic analysis software..."

Yes, we reworded it as you suggested.

**Line 446:** "was" should be "were"

Done. Thank you!
